# Supplementary material for: Serotonin signaling regulates actomyosin contractility during morphogenesis in evolutionarily divergent lineages
Source: Nat Commun. 2023 Sep 8;14:5547. doi: 10.1038/s41467-023-41178-w (PMC10491668; doi:10.1038/s41467-023-41178-w)
Supplement: Supplementary file 1 — Supplementary Information [file 41467_2023_41178_MOESM1_ESM.pdf]

Supplementary Information

Supplementary Figures

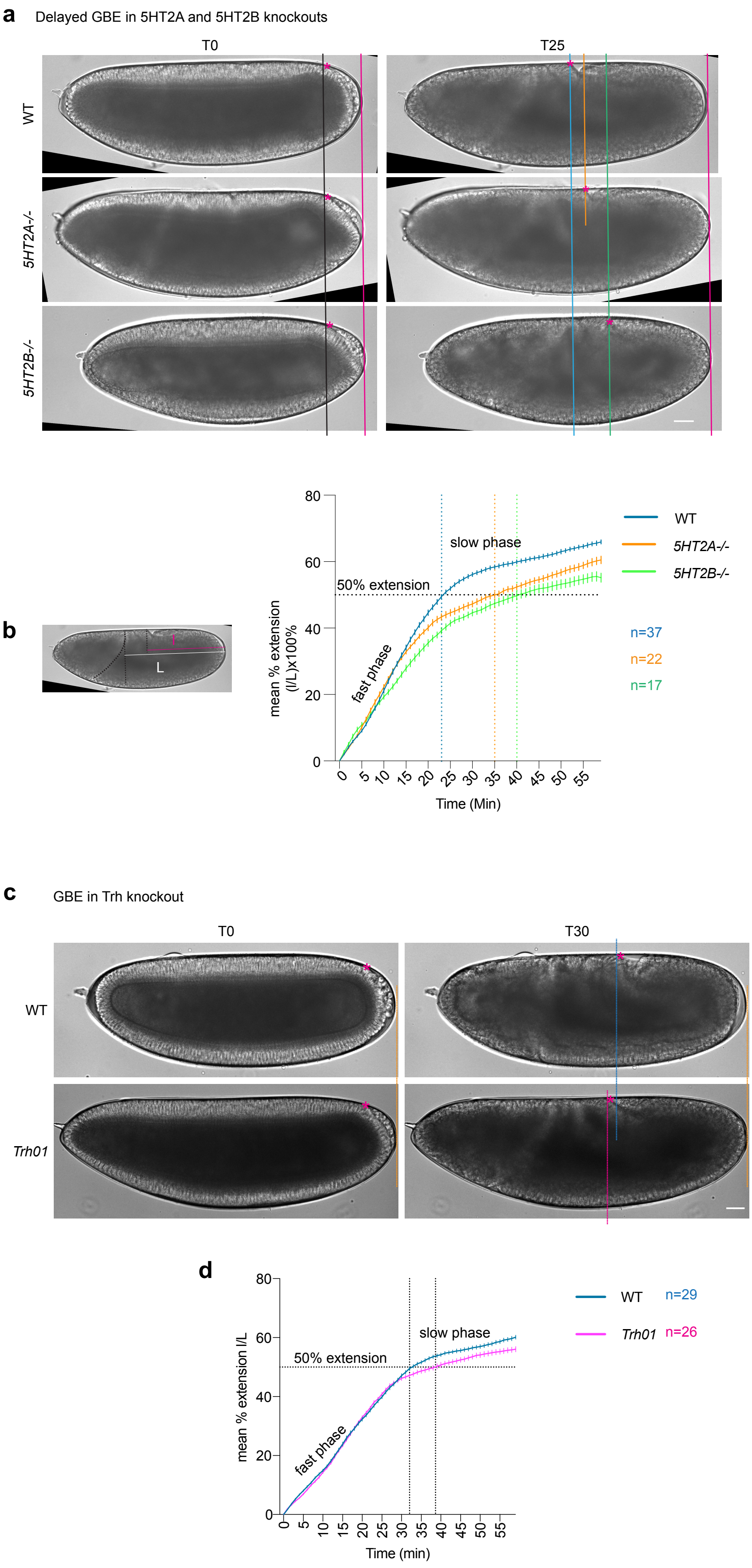

a
Delayed GBE in 5HT2A and 5HT2B knockouts
T0
T25
WT
5HT2A-/-
5HT2B-/-
b
l
L
80
60
40
20
0
mean % extension
(l/L)x100%
slow phase
50% extension
fast phase
0 5 10 15 20 25 30 35 40 45 50 55
Time (Min)
WT
5HT2A-/-
5HT2B-/-
n=37
n=22
n=17
c
GBE in Trh knockout
T0
T30
WT
Trh01
d
80
60
40
20
0
mean % extension l/L
slow phase
50% extension
fast phase
0 5 10 15 20 25 30 35 40 45 50 55
Time (min)
WT n=29
Trh01 n=26

Supplementary Fig. 1 | Germ band extension (GBE) in 5HT2A, 5HT2B and Trh knockout embryos.

a) Still DIC images taken at T0 and T25 min for wild type; WT (top panels), 5HT2A null mutant; *5HT2A-/-* (middle panels) and 5HT2B null mutant; *5HT2B-/-* (bottom panels). T0 is the onset of posterior midgut rotation. The point of contact of the tissue with the vitelline membrane, marked with the pink *, is tracked. **b**) Quantification of the average distance (± s.e.m) traversed by the contact point, normalized to the maximum length it can traverse. WT embryos take ~23 minutes to achieve 50% extension, while *5HT2A-/-* and *5HT2B-/-* null mutants take ~35 and ~40 minutes respectively. Error bars SEM. **c**) Still DIC images taken at T0 and T30 min for WT (top panels) and Trh^{01} null mutant (bottom panels). T0 is the onset of posterior midgut rotation. The point of contact of the tissue with the vitelline membrane, marked with the pink *, is tracked. **d**) Quantification of the average distance (± s.e.m) traversed by the contact point, normalized to the maximum length it can traverse. Scale bars 30 µm. Source data (**b** and **d**) are provided as a Source Data file.

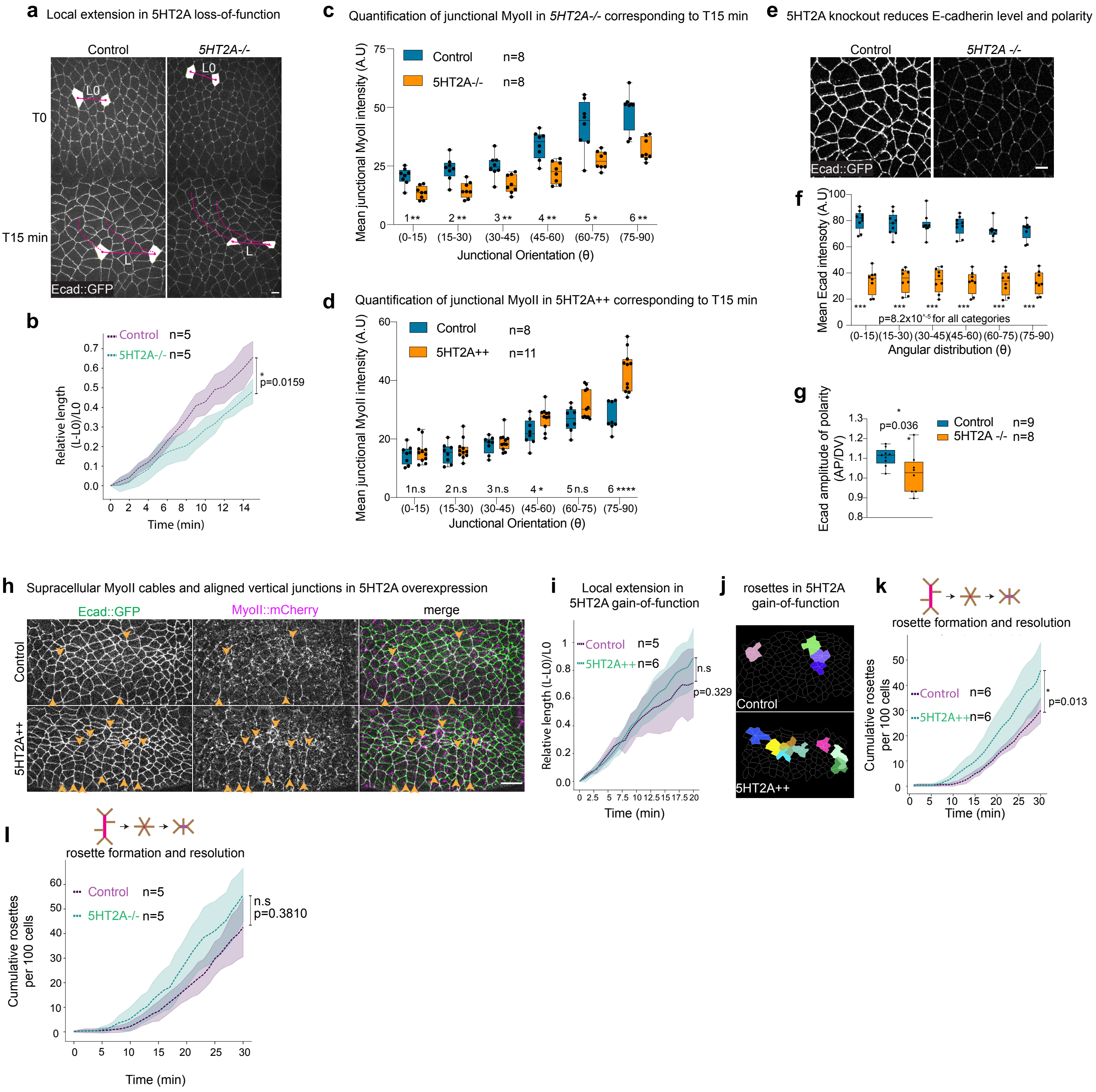

a Local extension in 5HT2A loss-of-function
Control
5HT2A-/-
T0
T15 min
Ecad::GFP
b
Control n=5
5HT2A-/- n=5
Relative length (L-L0)/L0
Time (min)
p=0.0159
c Quantification of junctional MyoII in 5HT2A-/- corresponding to T15 min
Control n=8
5HT2A-/- n=8
Mean junctional MyoII intensity (A.U)
Junctional Orientation (θ)
d Quantification of junctional MyoII in 5HT2A++ corresponding to T15 min
Control n=8
5HT2A++ n=11
Mean junctional MyoII intensity (A.U)
Junctional Orientation (θ)
e 5HT2A knockout reduces E-cadherin level and polarity
Control
5HT2A -/-
Ecad::GFP
f
Mean Ecad intensoty (A.U)
p=8.2x10^-5 for all categories
Angular distribution (θ)
g
Ecad amplitude of polarity (AP/DV)
p=0.036
Control n=9
5HT2A -/- n=8
h Supracellular MyoII cables and aligned vertical junctions in 5HT2A overexpression
Ecad::GFP
MyoII::mCherry
merge
Control
5HT2A++
i Local extension in 5HT2A gain-of-function
Control n=5
5HT2A++ n=6
n.s
p=0.329
Relative length (L-L0)/L0
Time (min)
j rosettes in 5HT2A gain-of-function
Control
5HT2A++
k
rosette formation and resolution
Control n=6
5HT2A++ n=6
p=0.013
Cumulative rosettes per 100 cells
Time (min)
l
rosette formation and resolution
Control n=5
5HT2A-/- n=5
n.s
p=0.3810
Cumulative rosettes per 100 cells
Time (min)

Supplementary Fig. 2 | Local tissue extension, rosettes formation, E-cad and MyoII distribution in 5HT2A loss-of-function and gain-of-funtion (Related to Fig. 1).

a) Local tissue extension in control (left panels) and 5HT2A null mutant; *5HT2A-/-* (right panels) at T0 (top panels) and T15 min (bottom panels). The centroid of the cells highlighted in white are tracked for 15 minutes. The dotted pink line represents the track of the centroid of the respective white cells. **b**) Quantification of the relative length (L-L0)/L0 in different genotypes. **c**) Quantification of MyoII intensities in different junctional orientation in control and *5HT2A-/-*. **d**) Quantification of MyoII intensities in different junctional orientation in control and 5HT2A++. (**e-g**); **e**) Ecad::GFP in control (left panel) and *5HT2A -/-* (right panel). Quantification of Ecad::GFP levels **f**), and polarity **g**) for the respective genotypes. p= 0.000082 in **f** for all junctional categories from two-tailed Mann-Whitney test. **h**) Snapshots of Ecad::GFP (left panel) and MyoII::mCherry (middle panel) and merge of both (right panel), in control (top panels) and 5HT2A overexpression, 5HT2A++ (bottom panels). Orange arrowheads indicate the aligned cell-cell junctions forming supracellular cables. **i**) Quantification of relative length (L-L0)/L0 in the respective genotypes. **j**) Representative images of rosettes in control (top panel) and 5HT2A++ (bottom panel) taken for T30 min. **k**) Quantification of cumulative rosette counts per 100 cells in the respective conditions. **l**) Quantification of cumulative rosette counts per 100 cells in control and 5HT2A null mutant, *5HT2A-/-*. The center dashed lines in **b, i, k** and **l** are the mean and the error bands are the standard deviation. In the box plots in **c**, **d**, **f**, and **g**, the line in the middle is plotted at the median. The box extends from the 25th to 75th percentiles. The whiskers indicate minimum and maximum values. Statistics: ns $p>0.05$, *$p\leq0.05$, **$p\leq0.005$, ***$p\leq0.0005$, ****$p\leq0.00005$ from (two-tailed) Mann-Whitney test.

p values in **c**: 1(p=0.002953), 2(p=0.001865), 3(p=0.001865), 4(p=0.001865), 5(p=0.006993), 6(p=0.002953).

p values in **d**: 1(p=0.657405), 2(p=0.657405), 3(p=0.777989), 4(p=0.040883), 5(p=0.090842), 6(p=2.6x10$^{\wedge-5}$).

n=number of embryos. Scale bars 5 µm. Source data (**b**, **c**, **d**, **f**, **g**, **i**, **k**, and **l**) are provided as a Source Data file.

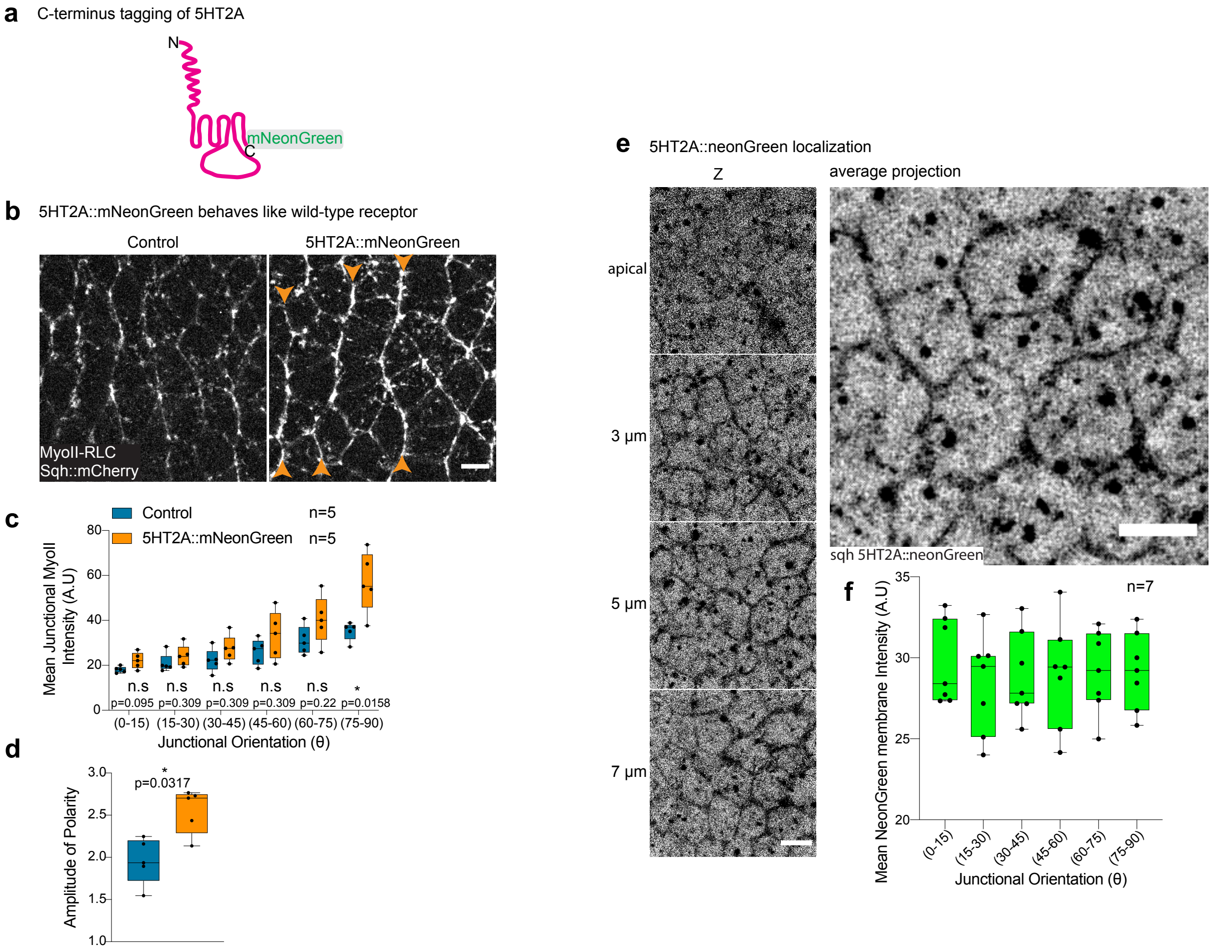

Supplementary Fig. 3 | Ectopic 5HT2A::mNeonGreen localization is not polarized yet MyoII is hyper-polarized.

a) Schematic showing C-terminal tagging of 5HT2A with mNeonGreen. (**b-d**) MyoII in 5HT2A::mNeonGreen. **b**) Still image of MyoII in control (left), 5HT2A::mNeonGreen over-expression (right, orange arrowheads indicate hyper-polarization of junctional MyoII). Quantification of junctional MyoII intensity distribution (**c**), and amplitude of polarity (**d**) in different conditions. (**e-f**) Localization of 5HT2A::mNeonGreen. Distribution of signal in different z-planes (left, top-bottom apical to basal), average projection (right) (**e**). Images in **e** are inverted (the dark signal is that of 5HT2A::mNeonGreen). Quantification of the distribution of membrane signal in different categories of junctions shows homogenous expression of the receptor (**f**). In the box plots in **c**, **d** and **f**, the line in the middle is plotted at the median. The box extends from the 25th to 75th percentiles. The whiskers indicate minimum and maximum values. Statistics: ns $p>0.05$, *$p\leq0.05$ from (two-tailed) Mann-Whitney test. n=number of embryos. Scale bars 5 µm. Source data (**c**, **d**, and **f**) are provided as a Source Data file.

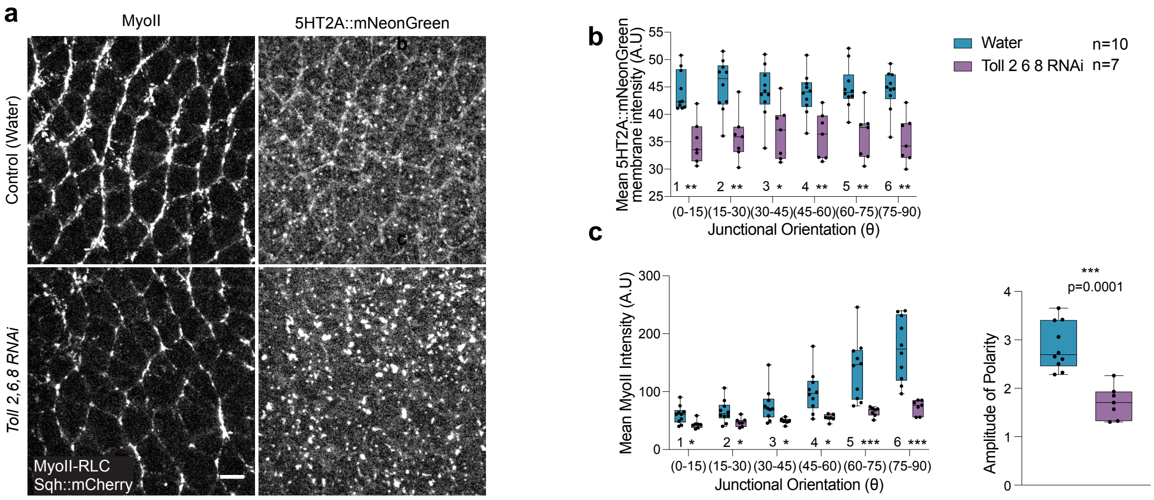

Supplementary Fig. 4 | 5HT2A::mNeonGreen distribution following Toll-2,6,8 knockdown.

(**a-c**) 5HT2A::mNeonGreen membrane localization following Toll-2,6,8 knockdown (*Toll-2,6,8 RNAi*). **a**) MyoII (top and bottom left panels) and 5HT2A::mNeonGreen (top and bottom right panels) images in water injected control (top panels) and *Toll 2,6,8 RNAi* injected embryos (bottom panels). Quantification of 5HT2A::mNeonGreen signal at the lateral membrane (**b**), and MyoII signal in different junction categories and amplitude of polarity (**c**) in respective conditions. In the box plots in **b** and **c**, the line in the middle is plotted at the median. The box extends from the 25th to 75th percentiles. The whiskers indicate minimum and maximum values. Statistics: ns $p>0.05$, *$p\leq0.05$, **$p\leq0.005$, ***$p\leq0.0005$ from two-tailed Mann-Whitney test.

p values in **b**: 1(p=0.001234), 2(p=0.003085), 3(p=0.009667), 4(p=0.001954), 5(p=0.001954), 6(p=0.001234).

p values in **c**: 1(p=0.018511), 2(p=0.033011), 3(p=0.006787), 4(p=0.006787), 5(p=0.000103), 6(p=0.000103).

n=number of embryos. Scale bar 5 μm. Source data (**b** and **c**) are provided as a Source Data file.

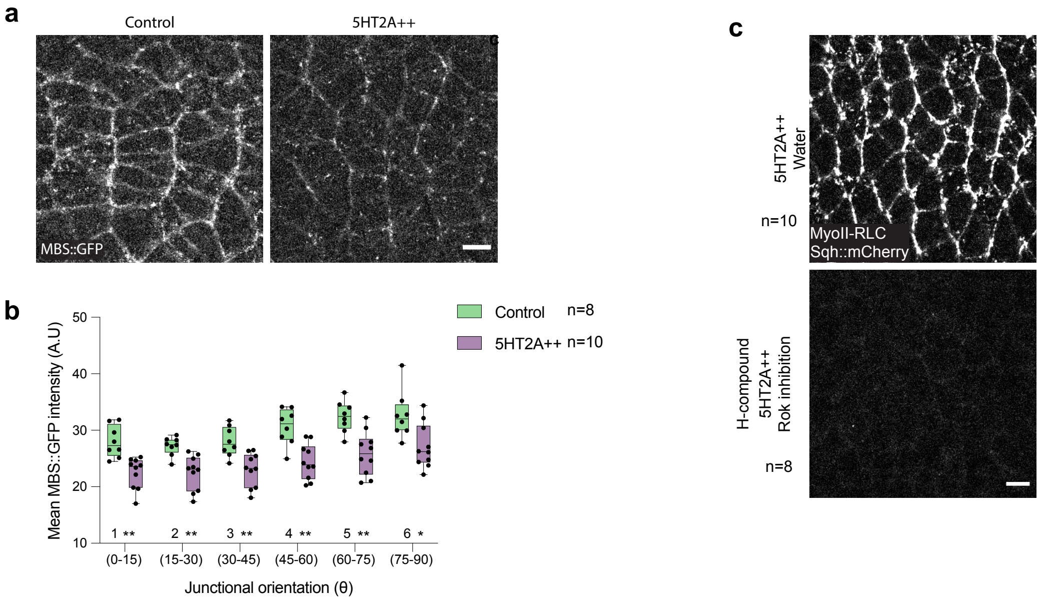

Supplementary Fig. 5 | Ectopic 5HT2A represses myosin-phosphatase and requires Rok activity to hyper-polarize junctional MyoII.

(**a-b**) Distribution of the myosin binding subunit (MBS::GFP) of myosin phosphatase in 5HT2A overexpression. Images of MBS::GFP in control (left panel) and 5HT2A++ (right panel) (**a**), quantification of junctional signal in respective conditions (**b**). (**c**) Rok inhibition with H-1152 compound in 5HT2A++. Snapshot of MyoII in water injected 5HT2A++ control (top panel) and H-compound injected 5HT2A++ embryo (bottom panel). Images in **c** are representative of n=10 water injected control and n=8 H-compound injected embryos. In the box plots in **b**, the line in the middle is plotted at the median. The box extends from the 25th to 75th percentiles. The whiskers indicate minimum and maximum values. Statistics in **b**: *$p \leq 0.05$, **$p \leq 0.005$ from two-tailed Mann-Whitney test. p values in **b**: 1(p=0.000548), 2(p=0.000868), 3(p=0.002057), 4(p=0.002057), 5(p=0.001371), 6(p=0.01554). n=number of embryos. Scale bars 5 µm. Source data (**b**) is provided as a Source Data file.

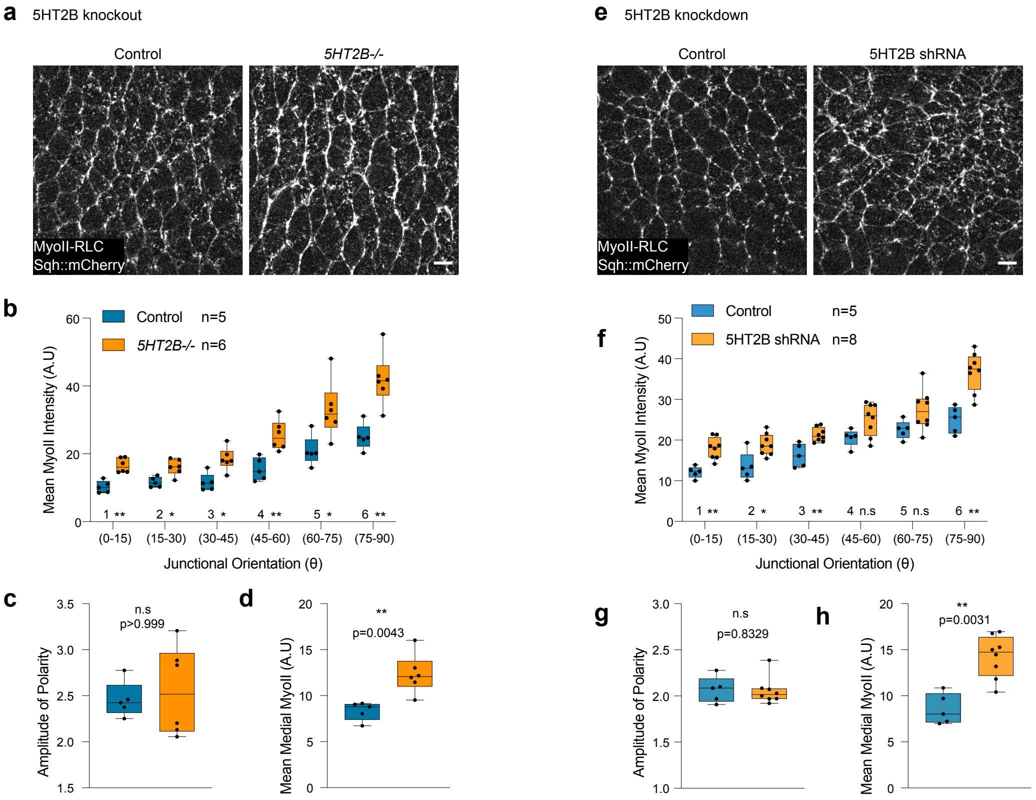

Supplementary Fig. 6 | MyoII distribution following 5HT2B knockout and knockdown. (**a-d**) MyoII distribution in 5HT2B null mutant (knockout) embryos. **a**) Snapshots of MyoII in control (left panel) and *5HT2B-/-* null mutant (right panel) embryos. Quantification of junctional MyoII intensities (**b**), amplitude of polarity (**c**), and medial MyoII intensities (**d**) in different genotypes. (**e-h**) MyoII distribution in 5HT2B knockdown embryos. **e**) Still images of MyoII in control (left panel) and 5HT2B shRNA overexpressing embryos (right panel). Quantification of junctional MyoII intensities (**f**), amplitude of polarity (**g**), and medial MyoII intensities (**h**) in respective conditions. In the box plots in **b**, **c**, **d**, **f**, **g** and **h**, the line in the middle is plotted at the median. The box extends from the 25th to 75th percentiles. The whiskers indicate minimum and maximum values. Statistics: ns $p>0.05$, *$p\leq0.05$, **$p\leq0.005$ from two-tailed Mann-Whitney test.

p values in **b**: 1(p=0.004329), 2(p=0.017316), 3(p=0.008658), 4(p=0.004329), 5(p=0.008658), 6(p=0.004329).

p values in **f**: 1(p=0.001554), 2(p=0.029526), 3(p=0.003108), 4(p=0.09324), 5(p=0.065268), 6(p=0.003108). n=number of embryos. Scale bars 5 µm. Source data (**b**, **c**, **d**, **f**, **g**, and **h**) is provided as a Source Data file.

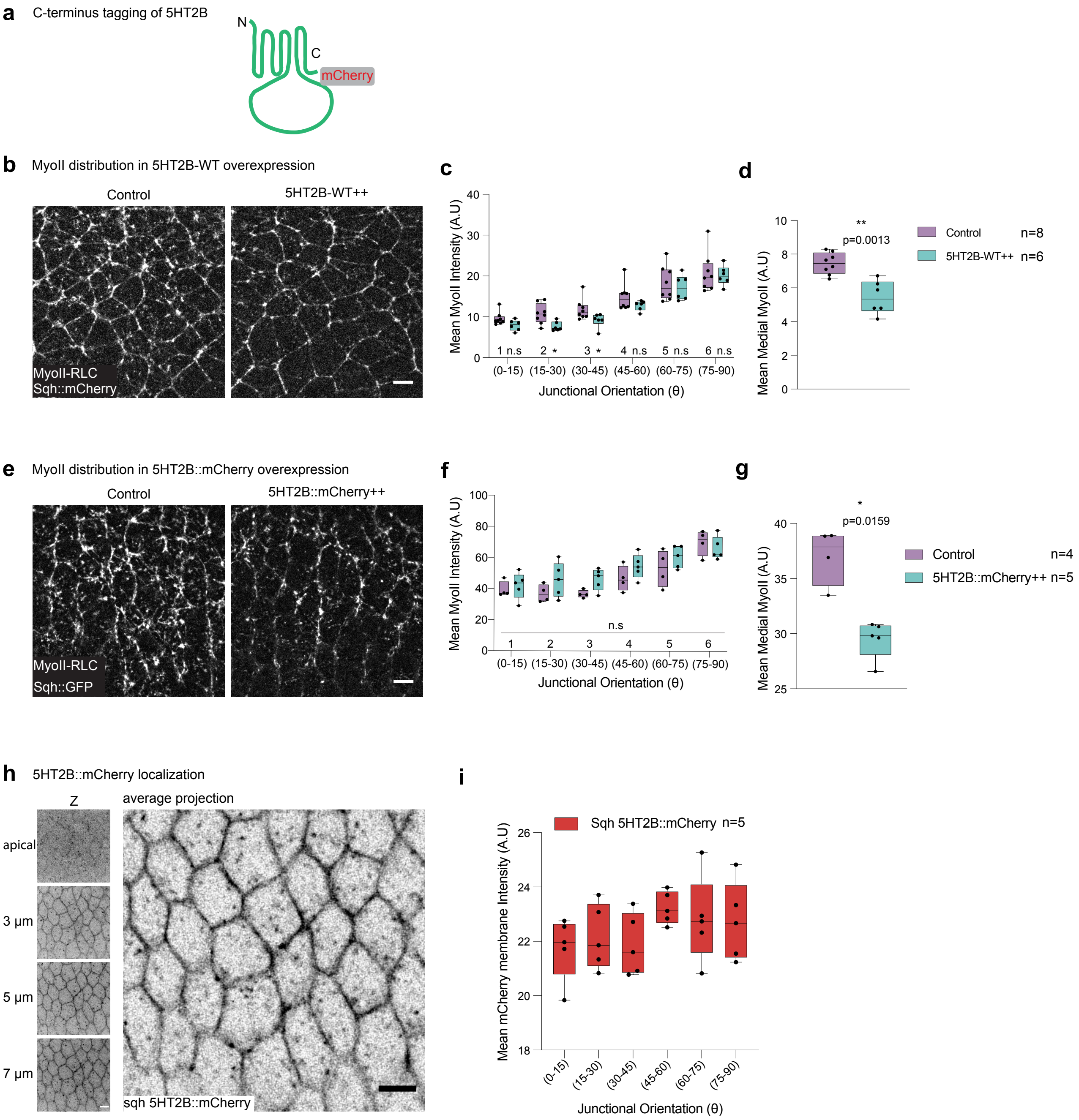
a C-terminus tagging of 5HT2B
N
C
mCherry
b MyoII distribution in 5HT2B-WT overexpression
Control
5HT2B-WT++
MyoII-RLC
Sqh::mCherry
c
Mean MyoII Intensity (A.U)
1 n.s 2 * 3 * 4 n.s 5 n.s 6 n.s
(0-15) (15-30) (30-45) (45-60) (60-75) (75-90)
Junctional Orientation (θ)
d
**
p=0.0013
Mean Medial MyoII (A.U)
Control n=8
5HT2B-WT++ n=6
e MyoII distribution in 5HT2B::mCherry overexpression
Control
5HT2B::mCherry++
MyoII-RLC
Sqh::GFP
f
Mean MyoII Intensity (A.U)
n.s
Junctional Orientation (θ)
g
*
p=0.0159
Mean Medial MyoII (A.U)
Control n=4
5HT2B::mCherry++ n=5
h 5HT2B::mCherry localization
Z
average projection
apical
3 µm
5 µm
7 µm
sqh 5HT2B::mCherry
i
Sqh 5HT2B::mCherry n=5
Mean mCherry membrane Intensity (A.U)
(0-15) (15-30) (30-45) (45-60) (60-75) (75-90)
Junctional Orientation (θ)

Supplementary Fig. 7 | Localization of 5HT2B::mCherry and MyoII distribution.

a) Schematic showing the C-terminus tagging of 5HT2B with mCherry. **b**) Distribution of MyoII in control (left panel) and 5HT2B-WT overexpressing (5HT2B-WT++) embryos (right panel). Quantification of MyoII at the junctions (**c**), and medial-apically (**d**) in different conditions. **e**) Images showing MyoII in control (left panel) overexpression of 5HT2B::mCherry (5HT2B::mCherry++) (right panel). Quantification of junctional (**f**), and medial MyoII intensities (**g**) in different conditions. **h**) Localization of the 5HT2B::mCherry in different z-planes (left, top-bottom apical to basal), average projection of the signal (right). Images in **h** are inverted (the dark signal is that of 5HT2B::mCherry). **i**) Quantification of 5HT2B::mCherry membrane signal. In the box plots in **c**, **d**, **f**, **g** and **i**, the line in the middle is plotted at the median. The box extends from the 25th to 75th percentiles. The whiskers indicate minimum and maximum values. Statistics: ns $p>0.05$, *$p\leq0.05$, **$p\leq0.005$ from (two-tailed) Mann-Whitney test.

p values in **c**: 1(p=0.107892), 2(p=0.012654), 3(p=0.029304), 4(p=0.344988), 5(p=0.754579), 6(p=0.949717).

p values in **f**: 1(p=0.555556), 2(p=0.285714), 3(p=0.111111), 4(p=0.285714), 5(p=0.285714), 6(p=0.730159).

n=number of embryos. Scale bars 5 µm. Source data (**c**, **d**, **f**, **g** and **i**) is provided as a Source Data file.

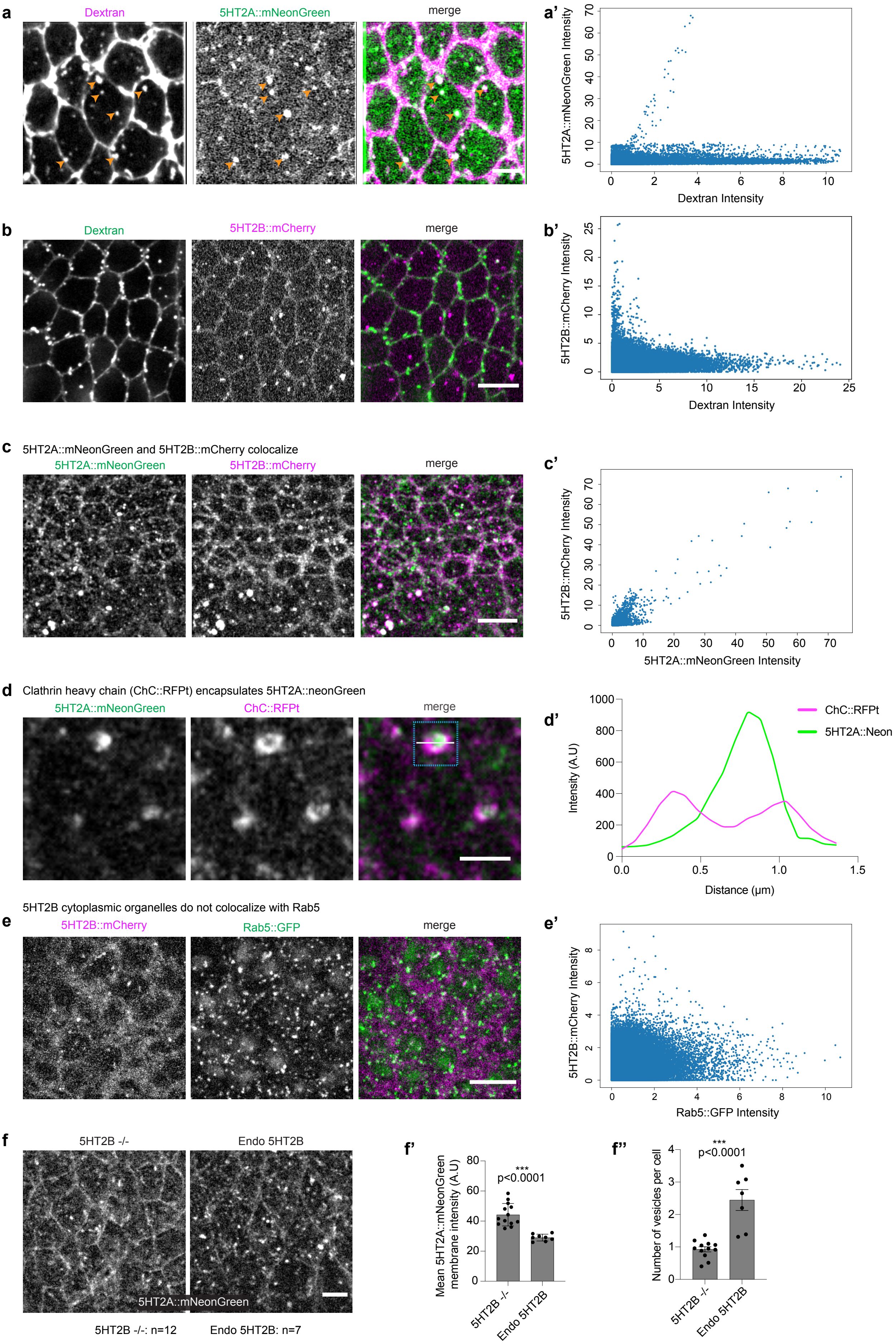

a
Dextran
5HT2A::mNeonGreen
merge
a'
5HT2A::mNeonGreen Intensity
Dextran Intensity
b
Dextran
5HT2B::mCherry
merge
b'
5HT2B::mCherry Intensity
Dextran Intensity
c
5HT2A::mNeonGreen and 5HT2B::mCherry colocalize
5HT2A::mNeonGreen
5HT2B::mCherry
merge
c'
5HT2B::mCherry Intensity
5HT2A::mNeonGreen Intensity
d
Clathrin heavy chain (ChC::RFPt) encapsulates 5HT2A::neonGreen
5HT2A::mNeonGreen
ChC::RFPt
merge
d'
Intensity (A.U)
ChC::RFPt
5HT2A::Neon
Distance (μm)
5HT2B cytoplasmic organelles do not colocalize with Rab5
e
5HT2B::mCherry
Rab5::GFP
merge
e'
5HT2B::mCherry Intensity
Rab5::GFP Intensity
f
5HT2B -/-
Endo 5HT2B
5HT2A::mNeonGreen
5HT2B -/-: n=12
Endo 5HT2B: n=7
f'
Mean 5HT2A::mNeonGreen membrane intensity (A.U)
***
p<0.0001
5HT2B -/-
Endo 5HT2B
f''
Number of vesicles per cell
***
p<0.0001
5HT2B -/-
Endo 5HT2B

Supplementary Fig. 8 | 5HT2B enhances 5HT2A endocytosis.

a) 5HT2A::mNeonGreen co-localizes with the dextran filled mobile cytoplasmic vesicles. Representative images of dextran (left), 5HT2A::mNeonGreen (middle) and merge (right). The orange arrowheads indicate the vesicles. Images are representative of 9 independent 5HT2A::mNeonGreen expressing embryos injected with dextran. (**a'**) Quantification of pixel-pixel intensity correlation between the two channels. **b**) 5HT2B::mCherry and dextran co-localization. Representative images of dextran (left), 5HT2B::mCherry (middle) and merge (right). Fewer 5HT2B::mCherry cytoplasmic organelles co-localize with dextran filled vesicles. Images are representative of 5 independent 5HT2B::mCherry expressing embryos injected with dextran. (**b'**) Quantification of pixel-pixel intensity correlation between the two channels. **c**) 5HT2A::mNeonGreen and 5HT2B::mCherry co-localize when co-expressed. Representative images of 5HT2A::mNeonGreen (left), 5HT2B::mCherry (middle) and merge (right). Images are representative of 12 independent embryos co-expressing 5HT2A::mNeonGreen and 5HT2B::mCherry. (**c'**) Quantification of pixel-pixel intensity correlation between two channels. **d**) 5HT2A::mNeonGreen organelles encapsulated in clathrin (ChC::RFPt) coated early endocytic organelle. Representative images of 5HT2A::mNeonGreen (left), ChC::RFPt (middle) and merge (right). Images are representative of 7 independent embryos co-expressing 5HT2A::mNeonGreen and ChC::RFPt. (**d'**) Line intensity plot along the white line in the vesicle highlighted in the blue box in the merge. **e**) 5HT2B::mCherry and Rab5::GFP marked early endosome does not co-localize. 5HT2B::mCherry (left), Rab5::GFP (middle) and merge (right). Images are representative of 6 independent embryos co-expressing Rab5::GFP and 5HT2B::mCherry. (**e'**) Quantification of pixel-pixel intensity correlation between the two channels. **f**) 5HT2A::mNeonGreen membrane levels and number of cytoplasmic organelles in 5HT2B null mutant (5HT2B-/-) embryos and embryos with endogenous levels of 5HT2B (Endo 5HT2B). Representative image of 5HT2A::mNeonGreen in 5HT2B-/- (left) and Endo 5HT2B (right). Quantification of 5HT2A::mNeonGreen membrane levels (**f'**) and the number of cytoplasmic organelles (**f''**), in different conditions. Data are representative of 7-12 embryos per condition as indicated. Cytoplasmic organelles were counted in 16-89 cells per embryo. Error bars SEM. Statistical significance calculated by (two-tailed) Mann-Whitney test (*** $p< 0.0005$). n=number of embryos. Scale bars in **a**, **b**, **c**, **e** and **f** 5 μm and in **d** 2 μm. Source data (**d'**, **f'** and **f''**) are provided as a Source Data file.

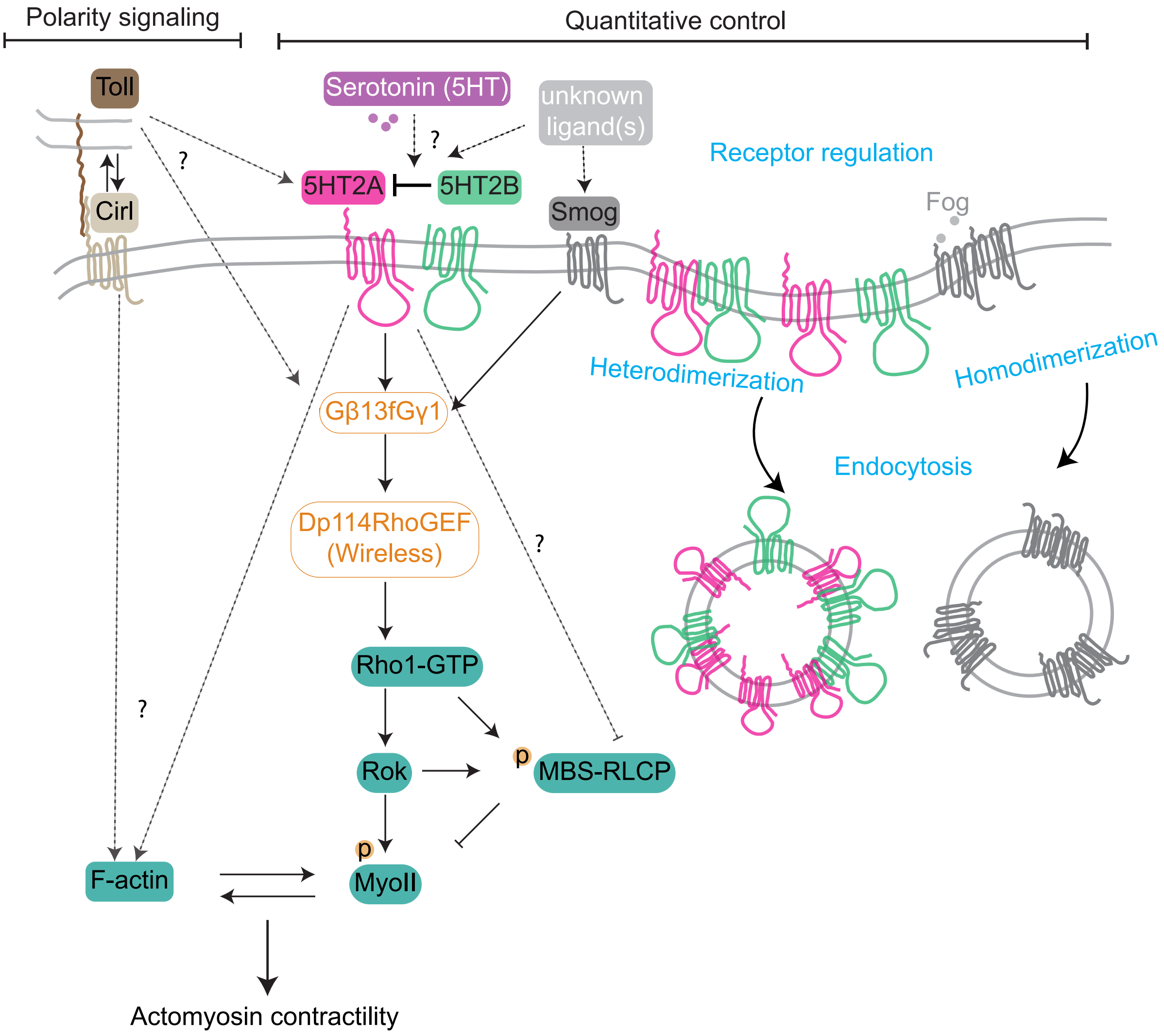

Supplementary Fig. 9 | Model.

Model showing the modular regulation of MyoII planar polarity by Toll receptors along with the GPCR Cirl and the quantitative control of Rho1/MyoII activation by GPCRs (serotonin receptors) signaling. Serotonin/5HT2A/5HT2B regulates the junctional MyoII levels through the Gβ13f/Gγ1, Dp114RhoGEF, and Rho1 signaling pathway and signals independently of the Cirl polarity signaling. 5HT2A has a multifunctional role and regulates F-actin, activates Rho1/MyoII and inhibits myosin-phosphatase. 5HT2B inhibits 5HT2A signaling through heterodimerization and subsequent endocytosis. Toll receptors polarizes Rho1 activity via unknown GPCR(s) and F-actin through interaction with the GPCR Cirl. Cirl does not activate Rho1, but regulates F-actin and thus MyoII. Smog signaling contributes to the junctional MyoII levels. Homodimerization (Smog) and heterodimerization (5HT2A/5HT2B) and subsequent endocytosis regulate the GPCRs membrane levels and signaling. The question marks (?) and dashed arrows indicate unknown mechanisms. MBS-RLCP: Myosin Binding Subunit of Myosin Regulatory Light Chain Phosphatase. Rok: Rho-associated kinase. MyoII: MyosinII. Cirl/Latrophilin.

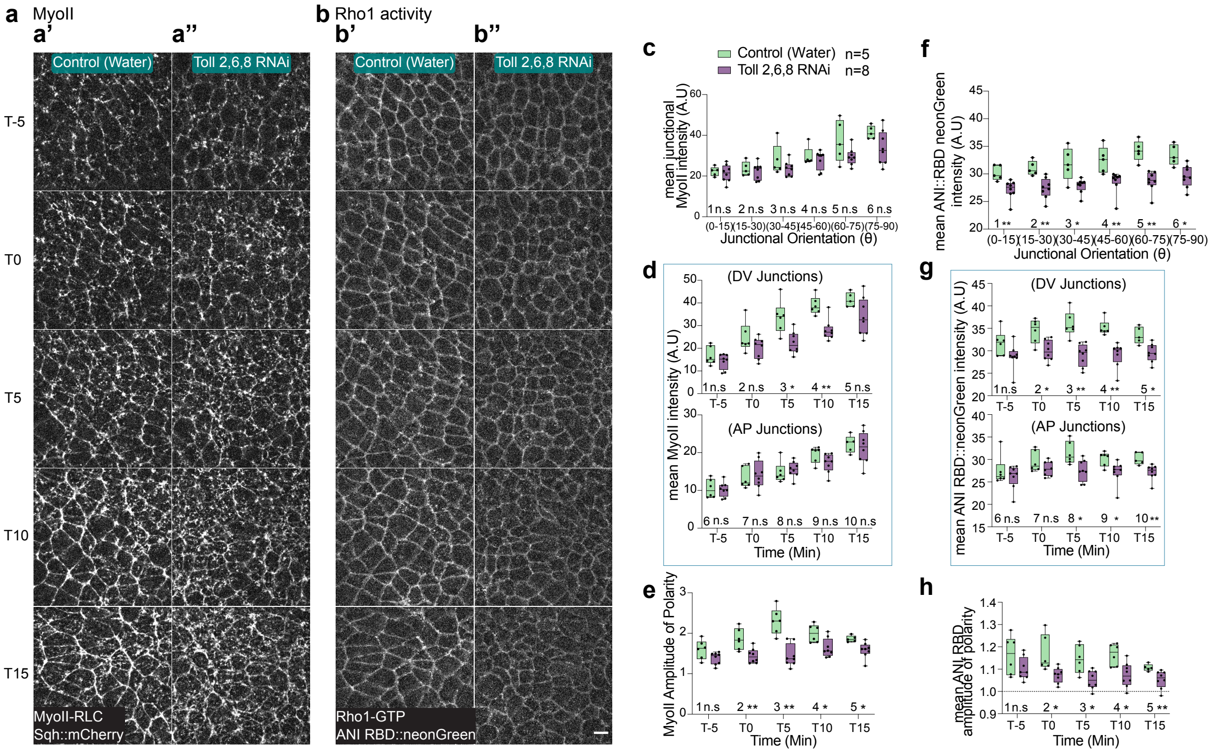

Supplementary Fig. 10 | Toll receptors polarizes MyoII by polarizing Rho1 activity. (**a-h**) MyoII (**a**), and Rho1-GTP biosensor (**b**) in Toll 2,6,8 triple knockdown over-time (from top to bottom). **a**) MyoII distribution in water injected control (**a'**) and Toll-2,6,8 dsRNA (*toll*-2,6,8 RNAi) injected embryos (**a''**) over time. **b**) Rho1-GTP biosensor signal distribution in control (**b'**) and Toll-2,6,8 dsRNA (*toll*-2,6,8 RNAi) (**b''**) over time. (**c-e**) Quantification of junctional MyoII distribution taken for T15 as an example (**c**), DV and AP oriented junctions over-time (**d**), and amplitude of polarity (**e**). (**f-h**) Quantification of Rho1-GTP biosensor signal for T15 as an example (**f**), DV and AP oriented junctions (**g**), and amplitude of polarity (**h**). In the box plots in **c**, **d**, **e**, **f**, **g** and **h**, the line in the middle is plotted at the median. The box extends from the 25th to 75th percentiles. The whiskers indicate minimum and maximum values. Statistics: ns p>0.05, *p≤0.05, **p≤0.005 from (two-tailed) Mann-Whitney test.

p values in **c**: 1(p=0.724165), 2(p=0.621601), 3(p=0.354312), 4(p=0.832945), 5(p=0.17094), 6(p=0.09324).

p values in **d**: 1(p=0.572761), 2(p=0.282384), 3(p=0.007992), 4(p=0.004662), 5(p=0.09324), 6(p=0.851815), 7(p=0.662005), 8(p=0.282384), 9(p=0.059274), 10(p=0.724165).

p values in **e**: 1(p=0.081252), 2(p=0.004662), 3(p=0.000666), 4(p=0.01998), 5(p=0.006216).

p values in **f**: 1(p=0.003108), 2(p=0.003108), 3(p=0.045066), 4(p=0.001554), 5(p=0.001554), 6(p=0.010878).

p values in **g**: 1(p=0.081252), 2(p=0.01998), 3(p=0.000666), 4(p=0.000666), 5(p=0.010878), 6(p=0.851815), 7(p=0.282384), 8(p=0.01998), 9(p=0.01998), 10(p=0.003108).

p values in **h**: 1(p=0.228438), 2(p=0.007992), 3(p=0.012654), 4(p=0.012654), 5(p=0.003108).

n=number of embryos. Scale bar 5 μm. Source data (**c**, **d**, **e**, **f**, **g**, and **h**) is provided as a Source Data file.

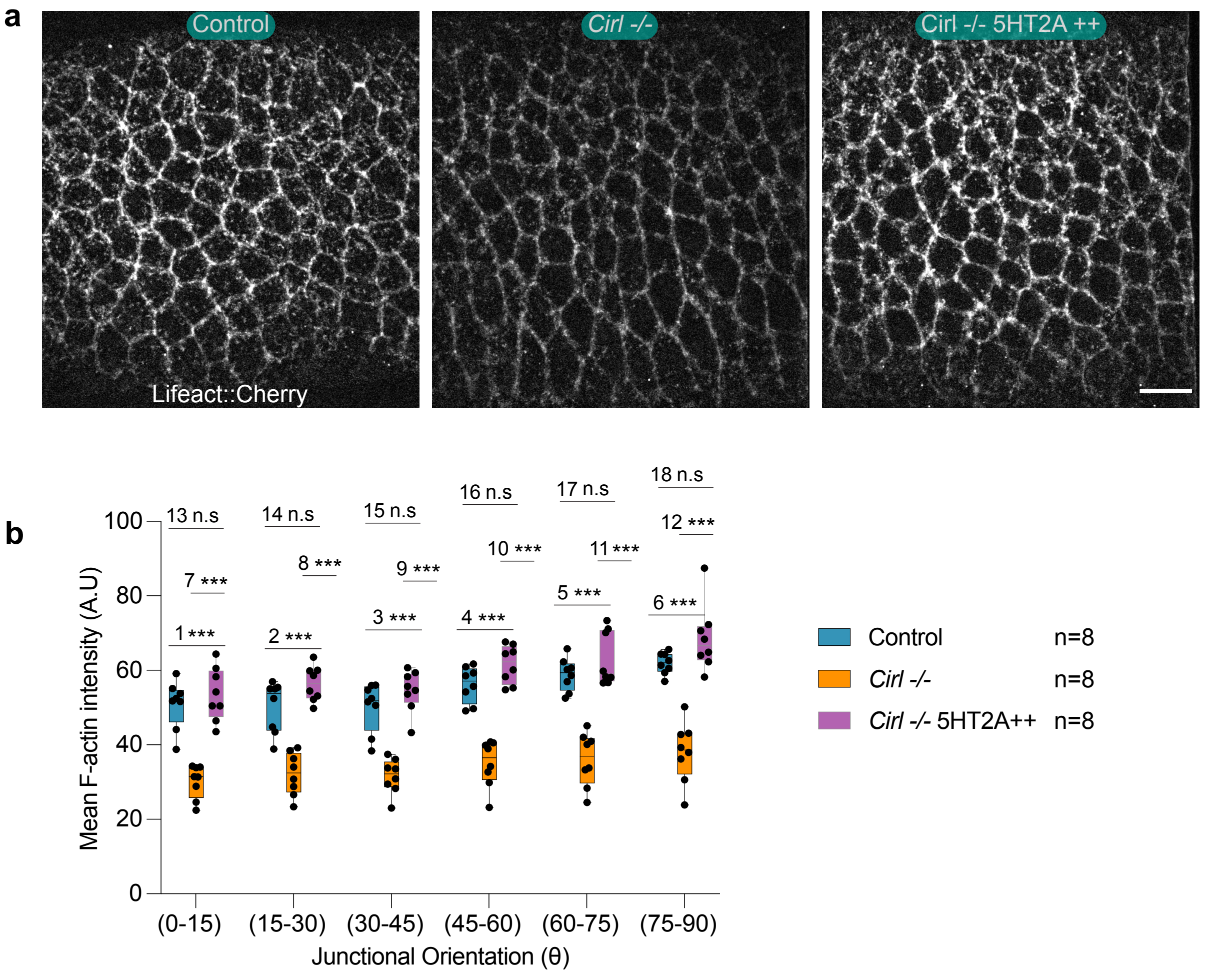

Supplementary Fig. 11 | 5HT2A and Cirl remodel F-actin.

a) Lifeact::mCherry still images in control (left panel), *Cirl -/-* (middle panel) and *Cirl-/-* and 5HT2A++ (right panel) embryos. **b**) Quantification of junctional F-actin levels in different conditions. In the box plots in **b**, the line in the middle is plotted at the median. The box extends from the 25th to 75th percentiles. The whiskers indicate minimum and maximum values. Statistics: ns p>0.05, ***p≤0.0005 from (two-tailed) Mann-Whitney test.

p values in **b**: 1(p=0.000155), 2(p=0.000311), 3(p=0.000155), 4(p=0.000155), 5(p=0.000155), 6(p=0.000155), 7(p=0.000155), 8(p=0.000155), 9(p=0.000155), 10(p=0.000155), 11(p=0.000155), 12(p=0.000155), 13(p=0.720901), 14(p=0.160528), 15(p=0.234499), 16(p=0.104895), 17(p=0.573737), 18(p=0.082984).

n=number of embryos. Scale bar 5 µm. Source data (**b**) is provided as a Source Data file.

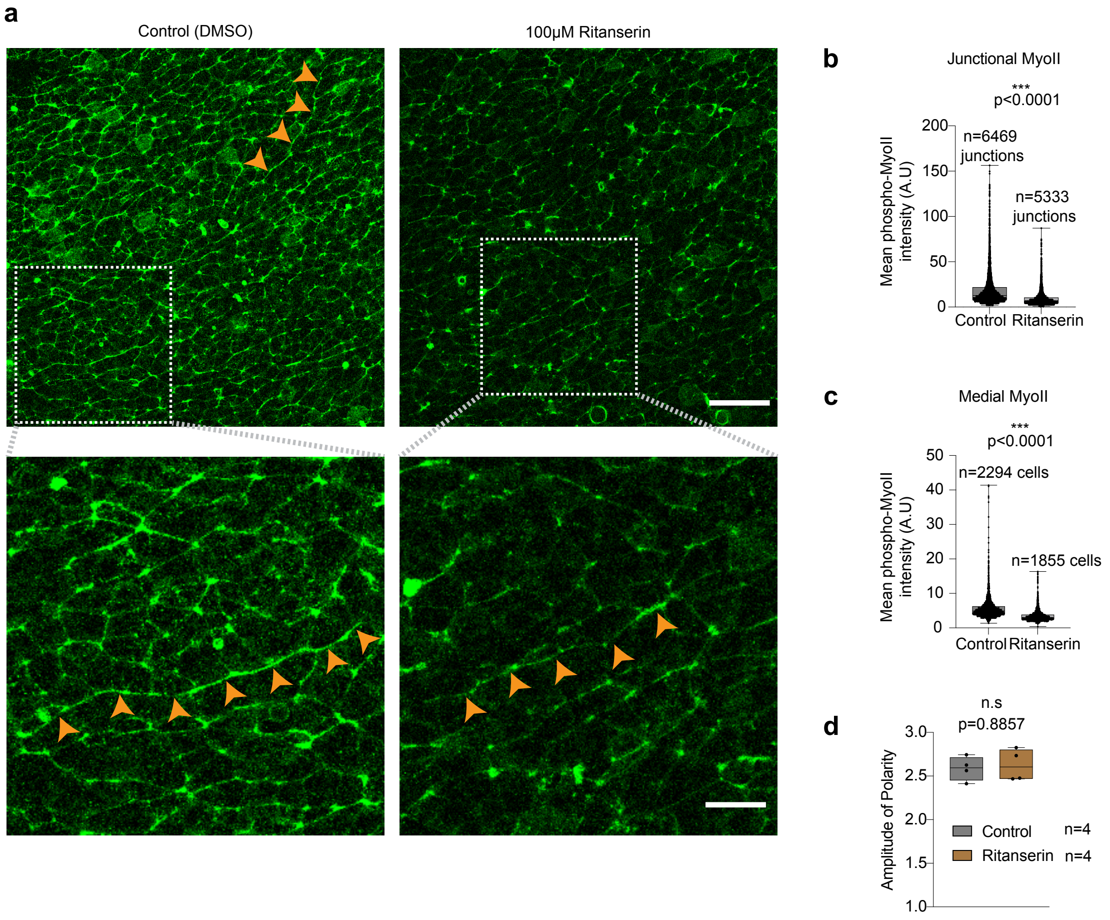

Supplementary Fig. 12 | Reduced MyoII activity after 5HT2A/2B antagonist treatment in chick embryo.

a) Representative images of phospho-MyoII in the embryo-proper (EP), contractile region in the DMSO treated control (left panel), 100 µM Retanserin treated (right panel) chick embryos. The white box in the images is magnified in the panels below each condition. The orange arrowheads indicate supracellular MyoII cables. Images are representative of at least 3 embryos per condition, experiments were replicated at least three times. The scale bar in the upper left panel is 50 µm, in the inset 20 µm. n= number of embryos. Quantification of junctional MyoII levels (**b**), medial MyoII levels (**c**), and amplitude of polarity (**d**) in different conditions. The plots in **b** and **c** are representative quantifications of the images in **a**. In the box plots in **b**, **c** and **d**, the line in the middle is plotted at the median. The box extends from the 25th to 75th percentiles. The whiskers indicate minimum and maximum values. Statistics: ns: p>0.05, **** p< 0.00005 from (two-tailed) Mann-Whitney test. Source data (**b**, **c** and **d**) is provided as a Source Data file.
