# Supplementary material for: Serotonin signaling regulates actomyosin contractility during morphogenesis in evolutionarily divergent lineages
Source: Nat Commun. 2023 Sep 8;14:5547. doi: 10.1038/s41467-023-41178-w (PMC10491668; doi:10.1038/s41467-023-41178-w)
Supplement: Supplementary file 3 — Description of Additional Supplementary Files [file 41467_2023_41178_MOESM3_ESM.pdf]

Description of Additional Supplementary Files

File Name: Supplementary Movie 1
Description: Differential Interference Contrast (DIC) time lapse of 5HT2A and 5HT2B null mutant embryos. WT (top panel), 5HT2A-/- (middle panel) and 5HT2B-/- (bottom panel). Images acquired every 1 min. A, Anterior; P, Posterior; D, Dorsal; V, Ventral.

File Name: Supplementary Movie 2
Description: MyoII dynamics in 5HT2A loss of function (5HT2A-/-). Live 100X imaging of MyoII (*sqh*::mCherry, left panels) and Ecad::GFP (right panels) in the ectoderm during germ-band extension. Top panels: Control; Bottom panels: 5HT2A null mutant, 5HT2A-/-. Video represents maximum projection of apical 10 planes spaced by 0.5 µm acquired every 15 sec.

File Name: Supplementary Movie 3
Description: MyoII dynamics in 5HT2A gain of function (5HT2A++). Live 100X imaging of MyoII (*sqh*::mCherry, left panels) and Ecad::GFP (right panels) in the ectoderm during germ-band extension. Top panels: Control; Bottom panels: 5HT2A over-expression, 5HT2A++. Video represents maximum projection of apical 10 planes spaced by 0.5 µm acquired every 15 sec.

File Name: Supplementary Movie 4
Description: MyoII dynamics in Trh loss of function (Trh^{01}). Live 100X imaging of MyoII (*sqh*::mCherry, left panels) and Ecad::GFP (right panels) in the ectoderm during germ-band extension. Top panels: Control; Bottom panels: Trh null mutant, Trh^{01}. Video represents maximum projection of apical 10 planes spaced by 0.5 µm acquired every 15 sec.

File Name: Supplementary Movie 5
Description: MyoII dynamics in Trh gain of function (Trh++). Live 100X imaging of MyoII (*sqh*::mCherry, left panels) and Ecad::GFP (right panels) in the ectoderm during germ-band extension. Top panels: Control; Bottom panels: Trh over-expression, Trh++. Video represents maximum projection of apical 10 planes spaced by 0.5 µm acquired every 15 sec.

File Name: Supplementary Movie 6
Description: MyoII dynamics in Trh knock-out and 5HT2A over-expression (Trh-/- 5HT2A++). Live 100X imaging of MyoII (*sqh*::mCherry, left panels) and Ecad::GFP (right panels) in the ectoderm during germ-band extension. Top panels: Control; Middle panels: 5HT2A++; Bottom panels: Trh-/- 5HT2A++. Video represents maximum projection of apical 10 planes spaced by 0.5 µm acquired every 15 sec.

File Name: Supplementary Movie 7
Description: MyoII dynamics in 5HT2A knock-out and Trh over-expression (*5HT2A*-/- Trh++). Live 100X imaging of MyoII (*sqh*::mCherry, left panels) and Ecad::GFP (right panels) in the ectoderm during germ-band extension. Top to bottom panels: Control; Trh++; *5HT2A*- /-; *5HT2A*-/-Trh++. Video represents maximum projection of apical 10 planes spaced by 0.5 µm acquired every 15 sec.

File Name: Supplementary Movie 8
Description: MyoII dynamics in Toll triple knock-down following 5HT2A over-expression. Live 100X imaging of MyoII (*sqh*::mCherry, left panels) and Ecad::GFP (right panels) in the ectoderm during germ-band extension. Top to bottom panels: Control; 5HT2A++; Toll-2,6,8RNAi; 5HT2A++ Toll-2,6,8RNAi. Video represents maximum projection of apical 10 planes spaced by 0.5 μm acquired every 15 sec.

File Name: Supplementary Movie 9
Description: MyoII dynamics in Cirl and 5HT2A genetic interaction. Live 100X imaging of MyoII (*sqh*::mCherry) in the ectoderm during germ-band extension. Top to bottom panels: Control; *5HT2A-/-; Cirl-/-; Cirl-/- 5HT2A*-/+. Video represents maximum projection of apical 10 planes spaced by 0.5 μm acquired every 15 sec.

File Name: Supplementary Movie 10
Description: F-actin and MyoII dynamics in Cirl knock-out and 5HT2A over-expression. Live 100X imaging of actin (Lifeact::mCherry, left panels) and MyoII (*sqh*::mCherry, right panels) in the ectoderm during germ-band extension. Top panels: Control; Middle panels: *Cirl- /-*; Bottom panels: *Cirl*-/- 5HT2A++. Video represents maximum projection of apical 10 planes spaced by 0.5 μm acquired every 15 sec.

File Name: Supplementary Movie 11
Description: Rho1-GTP biosensor dynamics in 5HT2A loss of function (5HT2A-/-). Live 100X imaging of Rho1 biosensor (ANI-RBD::mEGFP) in the ectoderm during germ-band extension. Top panel: Control; Bottom panel: *5HT2A*-/-. Video represents maximum projection of apical 10 planes spaced by 0.5 μm acquired every 1 min.

File Name: Supplementary Movie 12
Description: Rho1-GTP biosensor dynamics in 5HT2A gain of function (5HT2A++). Live 100X imaging of Rho1 biosensor (ANI-RBD::mEGFP) in the ectoderm during germ-band extension. Top panel: Control; Bottom panel: 5HT2A++. Video represents maximum projection of apical 10 planes spaced by 0.5 μm acquired every 1 min.

File Name: Supplementary Movie 13
Description: Myosin phosphatase dynamics following 5HT2A++. Live 100X imaging of myosin phosphatase (MBS::GFP) in the ectoderm during germ-band extension. Top panel: Control; Bottom panel: 5HT2A++. Video represents maximum projection of apical 10 planes spaced by 0.5 μm acquired every 30 sec.

File Name: Supplementary Movie 14
Description: MyoII dynamics in 5HT2B loss of function (*5HT2B-/-*). Live 100X imaging of MyoII (*sqh*::mCherry, left panels) and Ecad::GFP (right panels) in the ectoderm during germ-band extension. Top panels: Control; Bottom panels: 5HT2B null mutant, *5HT2B-/-*. Video represents maximum projection of apical 10 planes spaced by 0.5 μm acquired every 15 sec.

File Name: Supplementary Movie 15
Description: MyoII dynamics in 5HT2B knock-down. Live 100X imaging of MyoII

(*sqh*::mCherry, left panels) and Ecad::GFP (right panels) in the ectoderm during germ-band extension. Top panels: Control; Bottom panels: 5HT2B dsRNA. Video represents maximum projection of apical 10 planes spaced by 0.5 µm acquired every 15 sec.

File Name: Supplementary Movie 16
Description: MyoII dynamics in 5HT2A and 5HT2B double knock-down. Live 100X imaging of MyoII (*sqh*::mCherry, left panels) and Ecad::GFP (right panels) in the ectoderm during germ-band extension. Top to bottom panels: Control; 5HT2B dsRNA; *5HT2A-/-; 5HT2A-/-* 5HT2B dsRNA. Video represents maximum projection of apical 10 planes spaced by 0.5 µm acquired every 15 sec.

File Name: Supplementary Movie 17
Description: Rho1-GTP biosensor and MyoII dynamics following Toll-2,6,8 triple knock-down. Live 100X imaging of MyoII (*sqh*::mCherry, left panels) and Rho1-GTP biosensor (ANI-RBD::mNeonGreen) in the ectoderm during germ-band extension. Top panels: Control; Bottom panels: Toll-2,6,8 RNAi. Video represents maximum projection of apical 10 planes spaced by 0.5 µm acquired every 1min.

File Name: Supplementary Movie 18
Description: Brightfield time lapse of chick embryo treated with DMSO; Control (left panel) and Ritanserin 200 µM (right panel). Images acquired every 6 min.

File Name: Supplementary Movie 19
Description: Particle Image Velocimetry (PIV) time lapse of the chick embryo in Video 18. Left panel: Control (DMSO) and right panel: Ritanserin 200 µM. PIV analysis on consecutive images acquired every 6 min.

File Name: Supplementary Movie 20
Description: Top panels: brightfield time lapse of chick embryo treated with DMSO; Control (left panel) and Ritanserin 50 µM (right panel). Images acquired every 6 min.
Bottom panels: Particle Image Velocimetry (PIV) time lapse of the above embryos. Left panel: Control (DMSO) and right panel: Ritanserin 50 µM.
