# Supplementary material for: Serotonin signaling regulates actomyosin contractility during morphogenesis in evolutionarily divergent lineages
Source: Nat Commun. 2023 Sep 8;14:5547. doi: 10.1038/s41467-023-41178-w (PMC10491668; doi:10.1038/s41467-023-41178-w)
Supplement: Supplementary file 24 — Reporting Summary [file 41467_2023_41178_MOESM24_ESM.pdf]

nature portfolio

Corresponding author(s): Thomas Lecuit

Last updated by author(s): Jul 27, 2023

# Reporting Summary

Nature Portfolio wishes to improve the reproducibility of the work that we publish. This form provides structure for consistency and transparency in reporting. For further information on Nature Portfolio policies, see our Editorial Policies and the Editorial Policy Checklist.

## Statistics

For all statistical analyses, confirm that the following items are present in the figure legend, table legend, main text, or Methods section.

| n/a | Confirmed | |
|---|---|---|
| ☐ | ☒ | The exact sample size (*n*) for each experimental group/condition, given as a discrete number and unit of measurement |
| ☐ | ☒ | A statement on whether measurements were taken from distinct samples or whether the same sample was measured repeatedly |
| ☐ | ☒ | The statistical test(s) used AND whether they are one- or two-sided<br>*Only common tests should be described solely by name; describe more complex techniques in the Methods section.* |
| ☒ | ☐ | A description of all covariates tested |
| ☒ | ☐ | A description of any assumptions or corrections, such as tests of normality and adjustment for multiple comparisons |
| ☐ | ☒ | A full description of the statistical parameters including central tendency (e.g. means) or other basic estimates (e.g. regression coefficient) AND variation (e.g. standard deviation) or associated estimates of uncertainty (e.g. confidence intervals) |
| ☐ | ☒ | For null hypothesis testing, the test statistic (e.g. *F*, *t*, *r*) with confidence intervals, effect sizes, degrees of freedom and *P* value noted<br>*Give P values as exact values whenever suitable.* |
| ☒ | ☐ | For Bayesian analysis, information on the choice of priors and Markov chain Monte Carlo settings |
| ☒ | ☐ | For hierarchical and complex designs, identification of the appropriate level for tests and full reporting of outcomes |
| ☒ | ☐ | Estimates of effect sizes (e.g. Cohen's *d*, Pearson's *r*), indicating how they were calculated |

*Our web collection on statistics for biologists contains articles on many of the points above.*

## Software and code

Policy information about availability of computer code

| | |
|---|---|
| Data collection | Drosophila image acquisition was performed using Metamorph (V7.8.4) on Nikon dual camera Eclipse Ti inverted spinning disk microscope and with AxioVision V4.8 (brightfield imaging) on Zeiss inverted microscope. Zeiss, Zen 2.6 Pro (blue) for brightfield imaging of chick embryos and Zen 2.3Pro (black) on LSM-880 were used to image chick embryos. sFCCS data were collected using Zeiss Zen Black 2.3 SP1 FP3 (Service Pack 1 / Feature Pack 3) on LSM-880. |
| Data analysis | We used FIJI version 1.52, Tissue Analyzer V2.3 beta 2 (https://github.com/baigouy/tissue_analyzer), Manual Tracking (https://imagej.nih.gov/ij/plugins/track/track.html) for image analysis and GraphPad Prism 9 version 9.4.1 for data plotting and analysis. We have also developed custom python (3.7), JAVA, and MATLAB (R2020a) scripts to analyze the data. These codes are already published and have been cited appropriately in the manuscript (references 34, 51), however available on request from the corresponding author [T.L.]. sFCCS data analysis code is deposited in the GitHub (https://github.com/ValDunsing/ScanningFCCS). |

For manuscripts utilizing custom algorithms or software that are central to the research but not yet described in published literature, software must be made available to editors and reviewers. We strongly encourage code deposition in a community repository (e.g. GitHub). See the Nature Portfolio guidelines for submitting code & software for further information.

## Data

Policy information about availability of data

All manuscripts must include a data availability statement. This statement should provide the following information, where applicable:

- Accession codes, unique identifiers, or web links for publicly available datasets
- A description of any restrictions on data availability
- For clinical datasets or third party data, please ensure that the statement adheres to our policy

The data supporting the findings of this study and materials are available on request from the corresponding author [T.L.]. The Source Data file is provided in the paper.

## Human research participants

Policy information about studies involving human research participants and Sex and Gender in Research.

| | |
|---|---|
| Reporting on sex and gender | N/A |
| Population characteristics | N/A |
| Recruitment | N/A |
| Ethics oversight | N/A |

Note that full information on the approval of the study protocol must also be provided in the manuscript.

# Field-specific reporting

Please select the one below that is the best fit for your research. If you are not sure, read the appropriate sections before making your selection.

☒ Life sciences ☐ Behavioural & social sciences ☐ Ecological, evolutionary & environmental sciences

For a reference copy of the document with all sections, see nature.com/documents/nr-reporting-summary-flat.pdf

# Life sciences study design

All studies must disclose on these points even when the disclosure is negative.

| | |
|---|---|
| Sample size | No statistical method was used to predetermine sample size. The sample size was based on previous studies in the field (references 34, 37, 38, 39, 51, 65) and is indicated in the legends or next to the graph in the figures. |
| Data exclusions | Exclusion criteria were not pre-established. Embryos deemed as abnormal or damaged with respect to previously established criteria due to manipulation were omitted from analyses. |
| Replication | Drosophila quantifications were carried out on 4-37 independent experiments, each embryo was considered an independent experiment (for dsRNA injection, each mount was considered an independent experiment). All overexpression and knockdown genetic crosses were repeated at least three times. Phenotypes were consistent each time. Chick experiments were performed at least 3 times per condition on at least 3 embryos; each embryo was considered an independent experiment. All attempts at replication were successful to the extent reflected in the phenotypes, data distribution, and statistical tests described in the manuscript. In all experiments, we used appropriate controls, taking into account genetic backgrounds (please see 'Methods'), growth temperature, Myosin-II copy numbers (please see 'Methods'), and pharmacological treatments. |
| Randomization | The experiments were not randomized. Experimental groups were determined entirely by organism genotype or based on pharmacological treatment group. Identical growth temperature, imaging conditions and settings were used between the Drosophila experimental groups. Chick embryos (control and treated) were incubated at identical temperature in the same incubator and quantitative datasets were imaged together in one imaging session using identical imaging settings to minimize potential variation in imaging conditions. |
| Blinding | The investigators were not blinded to allocation during the experiments and outcome assessment due to the obvious phenotypes observed. |

# Reporting for specific materials, systems and methods

We require information from authors about some types of materials, experimental systems and methods used in many studies. Here, indicate whether each material, system or method listed is relevant to your study. If you are not sure if a list item applies to your research, read the appropriate section before selecting a response.

## Materials & experimental systems

| n/a | Involved in the study | |
|---|---|---|
| ☐ | ☒ | Antibodies |
| ☒ | ☐ | Eukaryotic cell lines |
| ☒ | ☐ | Palaeontology and archaeology |
| ☐ | ☒ | Animals and other organisms |
| ☒ | ☐ | Clinical data |
| ☒ | ☐ | Dual use research of concern |

## Methods

| n/a | Involved in the study | |
|---|---|---|
| ☒ | ☐ | ChIP-seq |
| ☒ | ☐ | Flow cytometry |
| ☒ | ☐ | MRI-based neuroimaging |

# Antibodies

**Antibodies used**

Antibodies were used for immuno-staining of chick embryos.

Primary antibodies used in this study were mouse anti-ZO1 at 1:250 dilution (Invitrogen ZO1-1A12) and rabbit anti-pMyosin light chain 2 at 1:50 dilution (Cell Signaling Technology CST-3671S and CST-3674S).

Secondary antibodies conjugated to AlexaFluor 488 or 555 were purchased from ThermoFisher Scientific (Goat anti-Rabbit IgG (H+L) superclonal™ Secondary Antibody, Alexa Fluor 488, Catalog Number A27034 and Goat anti-Mouse IgG (H+L) Superclonal™ Secondary Antibody, Alexa Fluor 555, Catalog Number A28180) and used at 1:500 dilutions.

**Validation**

The primary antibodies used in this study have been been validated and used extensively in various studies. For anti-ZO1 antibodies see https://www.thermofisher.com/antibody/product/ZO-1-Antibody-clone-ZO1-1A12-Monoclonal/33-9100 . This Antibody was verified by the supplier by Knockdown to ensure that the antibody binds to the antigen stated and published species include chicken.

Rabbit anti-pMyosin light chain 2 (Cell Signaling Technology CST-3671S): The antigen sequence used to produce this antibody shares 100% sequence homology with the chicken. Phospho-Myosin Light Chain 2 (Ser19) Antibody detects endogenous levels of myosin light chain 2 (smooth muscle) only when phosphorylated at serine 19. The antibody does not cross-react with the cardiac isoform of myosin light chain 2. See https://www.cellsignal.com/products/primary-antibodies/phospho-myosin-light-chain-2-ser19-antibody/3671.

Rabbit anti-pMyosin light chain 2 (Cell Signaling Technology CST-3674S): Phospho-Myosin Light Chain 2 (Thr18/Ser19) Antibody detects endogenous levels of myosin light chain 2 (smooth muscle) only when dually phosphorylated at threonine 18 and serine 19. The antibody does not cross-react with the cardiac isoform of myosin light chain 2. The antigen sequence used to produce this antibody shares 100% sequence homology with the chicken. See https://www.cellsignal.com/products/primary-antibodies/phospho-myosin-light-chain-2-thr18-ser19-antibody/3674

# Animals and other research organisms

Policy information about studies involving animals; ARRIVE guidelines recommended for reporting animal research, and Sex and Gender in Research

**Laboratory animals**

Drosophila Melanogaster and chick embryos were used in this study. All Drosophila experiments were carried out according to Campos-Ortega staging between stage 5 and stage 9. For chick experiments, embryo were used between HH1 and HH3 according to Hamburger Hamilton Stages.

**Wild animals**

Wild animals were not involved in this study.

**Reporting on sex**

The experimental procedure did not allow identifying the sex of the embryos.

**Field-collected samples**

This study did not involve field-collected samples.

**Ethics oversight**

No ethical approval or guidance was required.

Note that full information on the approval of the study protocol must also be provided in the manuscript.
